# Free‐Form Optical Fiber with a Square Mode and Top‐Hat Intensity Distribution

**DOI:** 10.1002/advs.202402886

**Published:** 2024-06-28

**Authors:** Rafal Kasztelanic, Hue Thi Nguyen, Dariusz Pysz, Hugo Thienpont, Takashige Omatsu, Ryszard Buczynski

**Affiliations:** ^1^ Faculty of Physics University of Warsaw Pasteur 5 Warsaw 02–093 Poland; ^2^ Lukasiewicz Research Network – Institute of Microelectronics and Photonics Wolczynska 133 Warsaw 01–919 Poland; ^3^ Department of Applied Physics and Photonics Vrije Universiteit Brussel Brussels Photonics Pleinlaan 2 Brussel 1050 Belgium; ^4^ Faculty of Engineering Chiba University 1–33 Yayoi‐cho Inage‐ku Chiba‐shi Chiba 263–8522 Japan

**Keywords:** effective medium theory, free‐form fibers, nanostructured fibers, optical fibers

## Abstract

The development of bend‐induced effectively single‐mode fiber with a square cross‐section and flat top‐hat intensity distribution is reported using core topology nanostructuring dedicated to femtosecond laser ablation systems. The fiber's core comprises 5419 silica and germanium‐doped silica nanorods with a diameter of 430 nm each arranged into a hexagonal lattice. The distribution of the rods is calculated using in‐house developed code based on the Monte Carlo algorithm to obtain a target shape of mode and intensity distribution. As a proof‐of‐concept, a silica nanostructured fiber with a 24 µm core is developed and verified against the purity of mode guidance, bending, and guiding losses. It is shown that for a wavelength of 1030 nm, the fiber is effectively single‐mode with 96% mode purity when bending with a radius of 20 cm is applied. The fiber has a measured mode area of 360 µm^2^, numerical aperture of 0.03, and total losses of 0.07 dB m^−1^.

## Graphical abstract

The development of an effectively single‐mode fiber with a square core and flat top‐hat intensity distribution is described using core topology nanostructurization. The core of the fiber is composed of thousands of silica and germanium‐doped silica nanorods arranged into a hexagonal lattice. As a proof of concept, a fiber with a 24 µm core is presented.

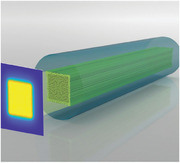

## 1 Introduction

Standard step‐index fibers, single and multimode, are characterized by a circularly symmetric core and a light energy distribution at the output similar to a Gaussian‐like distribution determined as Bessel of the first kind of zero order.^[^ ^1^ ^]^ Consequently, intensity distribution in the fundamental mode is Gaussian‐like, with maximum intensity on the optical axis and gradually decreasing with radius. No other technological approaches exist to radically modify the shape and energy distribution in single‐mode fibers. It should be noted that elliptical core fibers can be developed with standard modified chemical vapor deposition (MCVD), resulting in a two‐fold symmetry in the guided mode. However, the mode is always a solution of the characteristics equation,^[^ ^2^ ^]^ and only Gaussian‐like intensity distribution in the fundamental mode is obtained for any core cross sections.

For several years, we have observed an interest in finding an alternative design of optical fibers to modify the shape of intensity distribution toward a flat “top‐hat” one and turn the mode cross‐section into a square one. These efforts are motivated by research curiosity and applications such as femtosecond laser machining, high‐power beam delivery, photolithography, wafer processing, imaging, sensing, and spectroscopy.^[^ ^3^ ^]^

The flat intensity distribution is essential for high‐power lasers. First, a flat intensity profile is responsible for reducing the maximum energy density in the center of the mode and increasing the threshold level for fiber optical damage due to the dielectric breakdown in the core.^[^ ^4^, ^5^ ^]^ Second, the top‐hat distribution reduces the influence of nonlinear effects such as forced Brillouin scattering (SBS), forced Raman scattering (SRS), and self‐phase modulation (SPM).^[^ ^5^, ^6^ ^]^

Forming a square mode instead of a circular one is another challenge to address. Fibers with non‐circular, square mode are highly required for surface processing in laser machining.^[^ ^7^ ^]^ Solutions that offer uniform power distribution and square‐shaped beams are required for welding and surface multiple patterning (laser scribing^[^ ^8^ ^]^), waveguide forming, lithography direct writing, and in affecting biological tissues.^[^ ^9^ ^]^

### 1.1 Flat Intensity Distribution

Several approaches have been used to achieve a flat intensity distribution at the optical fiber output. First, there are optical systems based on refractive lenses^[^ ^10^ ^]^ and/or diffractive elements^[^ ^11^ ^]^ for beam shaping. Their disadvantage is the necessity of going to free space, macroscopic size, the necessity of alignment, and very high sensitivity to the alignment accuracy and dependence on the size of the input beam. Another approach is to modify the fiber (**Table** **1**). One possibility is etching the fiber's end to form a concave cone tip.^[^ ^12^ ^]^ The disadvantage of this solution is the need for wet chemistry. In addition, the intensity distribution is not uniform, and interfering with the front surface reduces the damage threshold of the optical fiber. Another possibility is to mix higher‐order modes, which have been used in long‐period grating approaches^[^ ^13^ ^]^ and on abrupt fiber tapering.^[^ ^14^ ^]^ In both cases, however, the beam profiles are very sensitive to the variation of bending. One can also mention multimode, multi‐trench fibers where it is possible to obtain a very large mode area (as a bend‐induced single mode fiber with an effective mode area of 1100 µm^2 [^ ^15^ ^]^) and thus a relatively flat intensity distribution in the central part of the fundamental mode.

**Table 1:** Review of approaches for optical fibers with top‐hat intensity and mode shaping.

No.	Fiber concept	Mode performance	Flat‐top diameter [um]	Mode cross‐section	Other properties	References
1.	Etching the end of the fiber to the concave cone tip	single‐ and multimode	200	Circular		[12]
2.	Long‐period grating	multimode	350	Circular	interference between LP_03_ and LP_04_ modes	[13]
3.	Abrupt taper	multimode	900	Circular		[14]
4.	Yb‐doped low index rod, silica microstructured cladding	multimode	30	Hexagon		[19]
5.	Square core jacketed air‐clad	multimode	400	Square		[7]
6.	Low index rod, high index ring, a complex array of submicrometer air‐holes in the cladding	single‐mode	12	nearly circular		[9]
7.	Low index rod, high index ring, a complex array of submicrometer air‐holes in the cladding	single‐mode	18.8	nearly circular	polarization‐maintaining	[18]
8.	Square porous core	multimode	240–420	Square	For THz applications	[20]
9.	Square M‐type fiber	multimode	44–82	Square		[23]

There have also been several attempts to develop a dedicated optical fiber with appropriate top‐hat intensity distribution.^[^ ^9^, ^16^, ^17^, ^18^, ^19^ ^]^ Examples include a multimode fiber with a Yb‐doped low‐index rod and silica microstructured cladding,^[^ ^19^ ^]^ multimode square core jacketed air‐clad fiber,^[^ ^7^ ^]^ and fiber with a square porous core.^[^ ^20^ ^]^ All the above solutions are based on multimode fibers and shape intensity distribution as a superposition of multiple modes. Indeed, this solution offers flat intensity. However, it is not practical for application in femtosecond ablation, since it provides a speckled structure with a high‐intensity dynamic sensitive to bending.

A very general strategy to improve the intensity flatness of the guided mode, where the core is surrounded by an additional high refractive index ring (M‐type fibers), was modeled by Elkin et al.^[^ ^21^ ^]^ However, technological limits, single‐mode performance, or experimental verification were not analyzed. A very similar approach for effective single‐mode fibers with relatively flattened intensity distribution based on a double air hole photonics lattice was reported by Valentin et al.^[^ ^9^ ^]^ and Gouriou et al.^[^ ^18^ ^]^ In both cases, the authors applied a double lattice of air holes to form a core and an additional central core with a lower refractive index to modify intensity distribution. As a result, a single‐mode fiber with mode purity over 15 dB is reported. The obtained uniform intensity distribution has a circular shape distorted at the edges by a hexagonal cladding structure. This approach is attractive but not very practical for implementation. It is challenging for robust mass fabrication (small air holes), difficult for efficient fusion splicing, and challenging for practical use (open air holes exposed to moisture and dust). To conclude, some solutions claim to offer a circular flattened intensity distribution. However, only the central part of the mode area is relatively flat.

### 1.2 Square Mode Shaping

The development of square core fibers was considered by Chow et al.,^[^ ^22^ ^]^ Habib et al.,^[^ ^20^ ^]^ and Hayes et al.^[^ ^7^ ^]^ However, the square core does not correspond to the square shape of guided modes, as shown by Habib et al. for THz range fibers.^[^ ^20^ ^]^ A relatively flat intensity distribution with multiple speckles was measured in fiber with the square core and air hole cladding with a very high filling factor (93%) fabricated by Hayes et al.^[^ ^7^ ^]^ Intensity flatness is a direct result of the superposition of massive multiple modes guided in the fiber, and the presence of speckles is related to mode interaction generated by a coherent laser source. Moreover, the square shape of the intensity distribution is disturbed at the edge by air holes. Such distortion is absent in the approach based on all‐glass multimode M‐type fibers.^[^ ^23^ ^]^ In this case, however, the problem is the reduction in beam intensity at the corners of the square core.

We can conclude that although there are several approaches, there is no solution for square mode top‐hat single‐mode or effectively single‐mode fibers that can be obtained with standard fiber technology, including the photonic crystal fiber approach. The state‐of‐the‐art results are summarized in Table 1.

**Figure 1:**
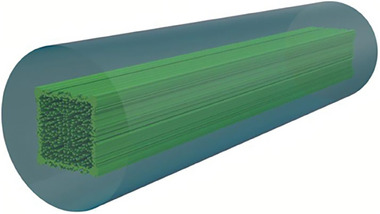
A free‐form nanostructured single‐mode fiber concept with square mode and top‐hat intensity distribution. A fiber core comprises a few thousand nanorods (with a diameter of 0.43 µm each) made of silica and germanium‐doped silica glass. The distribution of the rods forms an arbitrary free‐form refractive index distribution that determines shape and intensity distribution in the guided mode.

To find a new solution to this problem, we propose a radically new approach for optical fiber development based on core nanoengineering.^[^ ^24^, ^25^, ^26^, ^27^ ^]^ Nanostructuring allows the fabrication of all‐glass fibers (no air holes in the structure) and obtaining arbitrary effective refractive index distribution in the core (**Figure** **1**). The feasibility of this method was previously verified experimentally by fabricating a single‐mode fiber with a parabolic core and a fiber with high birefringence through artificially anisotropic glass to form the core.^[^ ^24^, ^25^ ^]^ Moreover, complex structures such as vortex beam converters or photosensitive and active fibers were developed with the same technology.^[^ ^26^, ^27^ ^]^

This paper reports on the design method, fabrication, and experimental verification of the effective single‐mode fiber guiding a fundamental mode with shaped spatial and intensity profile distribution. The main objective was to obtain a fiber that, on the one hand, guides the fundamental mode with a shape as close as possible to a square top‐hat function and, on the other hand, was characterized by a large effective mode area and maintains a single‐mode performance simultaneously. Our motivation for research has several aspects. First of all, we try to verify if we can obtain all‐solid silica fiber with arbitrarily designed mode properties based on the core nanostructuring approach and using standard silica‐based glass components developed with MCVD technology and stack and draw preform assembly technique (typical for the development of photonic crystal fibers and imaging bundles). Developing a fiber dedicated to femtosecond laser ablation is our second motivation. A compact all‐fiber tool without external optics would help ablate tiny micron‐sized features with sharp edges and flat surfaces. This experiment also verifies the ability of nanostructuring to develop top‐hat large‐mode area fibers for high‐power beam delivery. In this case, the square mode is a redundant property since a circular cross‐section would be more suited for this particular application.

## 2 Design of Square‐Core Optical Fiber

### 2.1 Numerical Analysis of Optical Fibers with a Square Core

**Figure 2:**
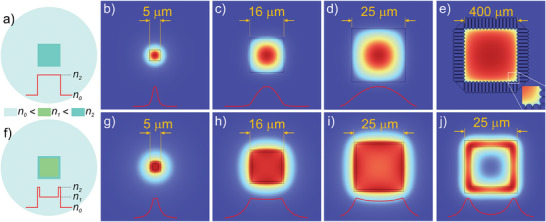
Intensity distribution in optical fibers with square‐like cores: a) step‐index fiber with a simple square core, b–d) intensity distribution in the fundamental mode in step‐index fiber for fibers with 5, 16, and 25 µm core size respectively; e) calculated intensity distribution in multimode fiber with large core and air cladding, as proposed by Hayes et al.^[^ ^7^ ^]^; f) M‐type fiber; g–i) intensity distribution in the fundamental mode in M‐type fiber for fibers with 5, 16, and 25 µm core size, respectively; j) an example of mode localization in M‐type fiber with the thick frame. Modeling was carried out for a frame thickness of 3 µm, wavelength λ = 1.03 µm, and refractive indices: n0 = 1.450, n1 = 1.451, n2 = 1.453, typical for silica‐doped glass fibers.

To illustrate the dependence of the shape of the fiber core on the intensity distribution in the guided modes we have performed simulations with the finite elements method (FEM) and an eigenvalue mode solver using the Comsol Multiphysics software environment. We introduce the geometrical and material parameters of the fiber, and with an eigenvalue solver, we obtain a set of steady‐state solutions. The set is further analyzed to determine the parameters of guided modes in the considered fiber structure. This is the typical approach for analyzing fibers with arbitrary cross‐sections.^[^ ^28^ ^]^ As described in the introduction, a simple change in the shape of a circular step‐index core to a square core (**Figure** **2**a) does not lead to a top‐hat square‐shaped fundamental mode. When the diameter of the core is increased, the mode intensity profile is slightly flattened at the edges but very similar to a Gaussian mode (Figure 2b–d). Obtaining a uniform intensity distribution at the output of a square‐core fiber is possible when fibers with large cores are used, where massively multiple modes are involved in forming the final intensity distribution (Figure 2e). In this case, we applied an additional ring of air holes in the cladding, as proposed in,^[^ ^7^ ^]^ to limit the mode area and uniformly distributed intensity to the core region, which may cause irregularities at the edges of the modes like “postage stamp”.

A significant improvement in terms of uniformity of light intensity distribution in the fundamental mode is obtained using M‐type all‐glass optical fiber (Figure 2f). In this type of fiber, the square core is supplemented with a narrow glass frame with an increased refractive index (Figure 2g–i). By selecting the appropriate frame thickness for a given core size and the refractive indices of the frame and the area within the frame, a relatively flat top‐hat intensity distribution in the center of the fundamental mode can be obtained (Figure 2h). Simultaneously, a flattened center of the mode is surrounded by an area where a monotonic intensity decrease is observed (≈45% of the total effective mode area). The frame with a higher refractive index ensures that some of the energy of the fundamental mode is redistributed and located inside this frame at the cost of energy from the center of the core, where the refractive index is lower. Nevertheless, this approach is very sensitive to wavelength and bending. An improper selection of the refractive index of the individual elements of such fiber and/or the use of too thick a frame can lead to the selective localization of energy in the frame region (Figure 2j).

**Figure 3:**
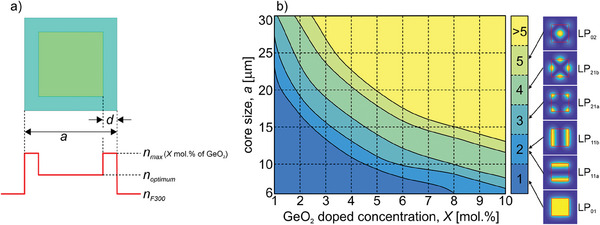
Analysis of guided mode population in M‐type fibers: a) parameters of the considered fiber, b) dependence of the number of guided modes on the core size *a*, and dopant concentration *X* in the frame with increased refractive index.

Since one of the main objectives of the work was to design a fiber allowing for an efficient single‐mode operation,^[^ ^2^ ^]^ in the next stage, we analyze how the number of guided modes in an M‐type fiber depends on core size *a*, and the GeO_2_ doping level *X*, in the frame surrounding the core (**Figure** **3**a). In this analysis, we consider only optimum solutions where the width of the frame *d*, and doping of the central part of the core are selected to obtain the flattest intensity distribution in the fundamental mode. The results, presented in Figure 3b, show that M‐type fibers offer mainly multimode solutions, and the modes are similar to those guided in buried channel waveguides.^[^ ^29^ ^]^ We note that since we do not maintain circular symmetry of the core numerical solutions corresponding to Hermite–Gaussian‐like transverse electromagnetic modes (TEM), intensity distribution differs from analytical solutions obtained for simple square core waveguide or circular core fibers.^[^ ^30^, ^31^ ^]^ The presented modes have discrete eigenvalues and their intensity distribution corresponds to eigenvectors calculated as direct numerical solutions of wave equation for eigenmodes in specific border conditions of M‐type fibers. We have used Finite Elements Method (FEM) to find eigenmodes. For simplicity, we will further refer to calculated TEMxy modes as linearly polarized modes LPxy to maintain standard fiber optics nomenclature.

The maximum core size for single‐mode fibers does not exceed 20 µm (corresponding to an effective mode area of 330 µm^2^), even at low doping of 1%. Moreover, this solution is very sensitive to bending, which significantly deforms the square shape of the mode. Higher‐order modes (HOMs) appear for higher doping levels and for increased core size. Therefore, considering the practical use of the M‐type fibers, fibers with a maximum core of 25 × 25 µm may offer a few‐mode operation and bend‐induced single‐mode performance. We note that this structure can only be considered a theoretical solution since MCVD cannot be applied to develop considered rotational asymmetric core geometry.

An in‐depth analysis of the fundamental mode intensity distribution in M‐type fibers shows that even with optimally chosen parameters such as refractive index, frame thickness, and core size, the mode has a rectangular shape with rounded corners and reduced intensity at the corners (Figure 2h,i). Further modification of core geometry is required to obtain a truly top‐hat square intensity distribution in the fundamental mode. In particular, intensity redistribution is needed to balance low intensity in the corners of the mode. This redistribution can be done only using the nanostructuring approach.

### 2.2 A Concept of Nanostructured Medium for Free‐Form Fibers

**Figure 4:**
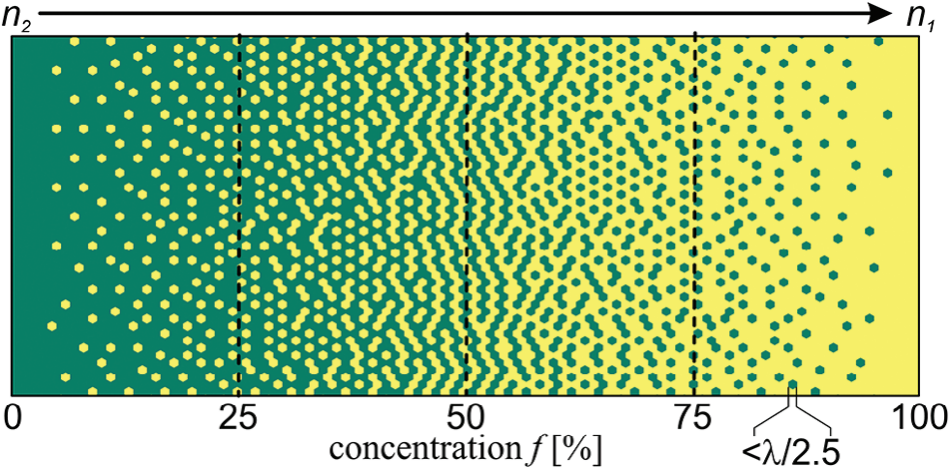
Example of a nanostructured material with the linear decrease of concentration *f* and effective refractive index from n_2_ to n_1_, assuming the maximum feature size condition is satisfied.

The basis of the nanostructuring technique is the concept of effective medium, which is described by the Maxwell‐Garnett effective medium approximation model (EMA).^[^ ^32^ ^]^ In this approach, the physical properties of a dielectric material composed of discrete rods with different physical properties are assumed to be a weighted average of the refractive indices of the individual rods in a local neighborhood. In the most straightforward approach, using only two types of rods, an effective refractive index *n_eff_* can be calculated locally as:^[^ ^32^ ^]^

(1) $$n_{eff}^{2}=fn_{1}^{2}+\left(1-f\right)n_{2}^{2}$$

where *f* determines the local concentration of the first glass with refractive index *n_1_*, and *n_2_* denotes the refractive index of the second component, respectively. A maximum feature size condition must be satisfied for individual rods to consider multiple rods structure as an effective medium. As we have verified experimentally in our previous works, the effective medium condition is fulfilled for the diameter of individual nanorods of the order of *λ*/2.5 or smaller.^[^ ^33^ ^]^ The nanostructuring allows for arbitrary effective refractive index distribution within the limits determined by the materials used. An example of an effective medium with a continuously linear change of concentration f and effective refractive index from n_2_ to n_1_ is shown in **Figure** **4**, where every spot denotes a nanorod in a vertical position. Such structures can be produced by stacking a preform of two types of glass rods and then rescaled and integrated by drawing it on the standard optical drawing tower so that the final structure meets the maximum feature size condition.^[^ ^24^ ^]^

The advantage of using nanostructuring for the design and fabrication of optical fibers is that the several parameters that may conflict with each other can be optimized in parallel, as in the case of few‐mode fibers, where the number of guided modes and maximum distance between their propagation constants are optimized.^[^ ^34^ ^]^ As a result, it is possible to achieve a trade‐off between different desired fiber features.

### 2.3 Determination of Optical Properties of Nanorods for Free‐Form Fiber Development

Developing fibers with nanostructured cores requires precise control of the refractive index distribution within the core. Therefore, the optical properties of nanorods have to be determined and used as a part of the design process. In the considered fiber, we assume that the optical fiber cladding and low refractive index nanorods are made of silica glass F300 (Heraeus). The high refractive index nanorods are made of silica doped with GeO_2_. The material dispersion of both types of glasses can be estimated using a modified formula proposed by Fleming.^[^ ^35^ ^]^

### 2.4 Numerical Optimization of the Refractive Index Distribution in the Free‐Form Fiber

**Figure 5:**
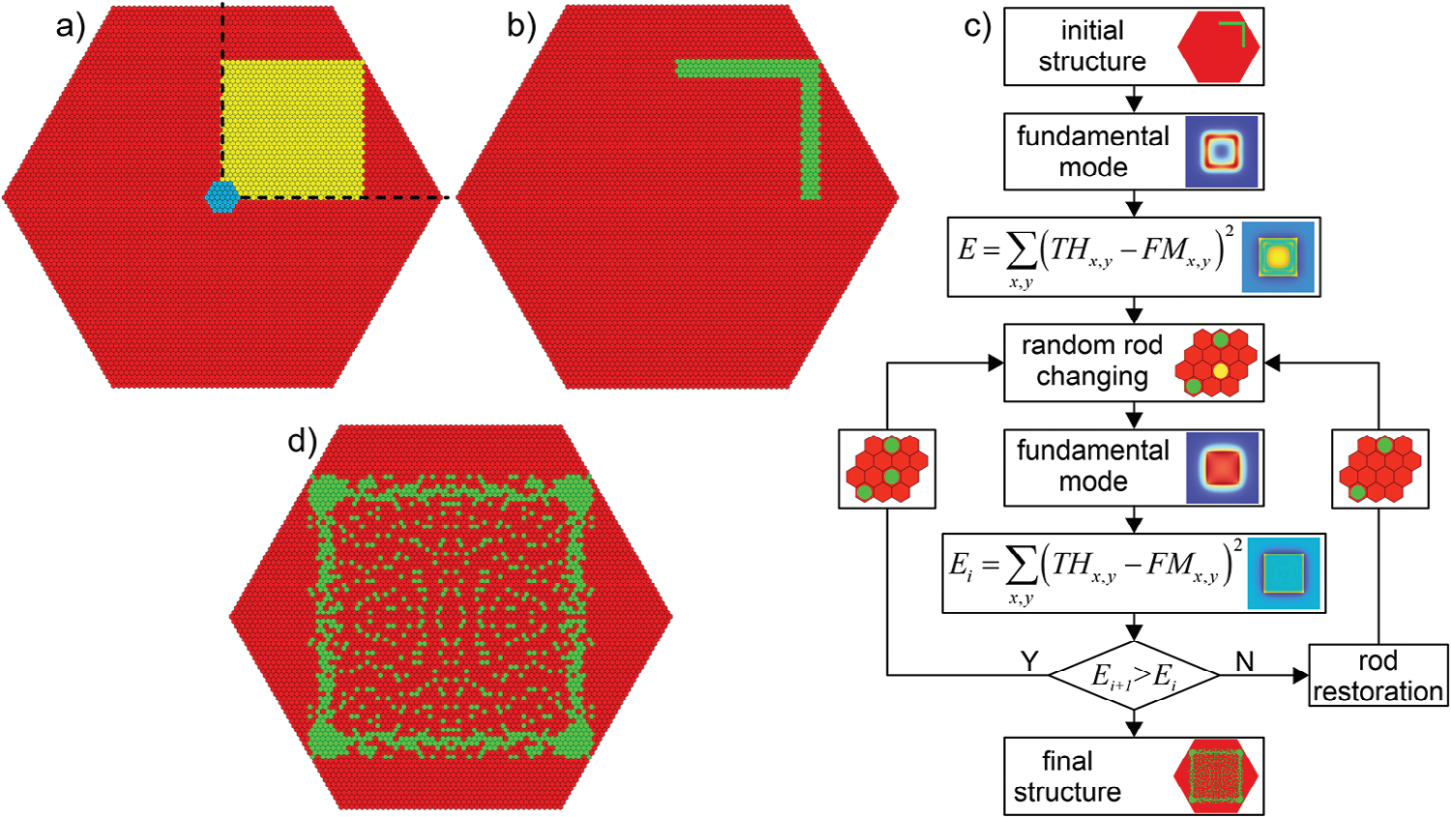
A scheme of the algorithm for optimization of nanorods distribution in nanostructured core composed of silica (red) and germanium (green) doped silica rods. a) core areas for optimization, b) initial distribution of high refractive index rods, c) schematic of optimization algorithm (the randomly changed rod is highlighted in yellow), d) calculated optimum structure of the core for guiding fundamental mode with square, top‐hat intensity distribution.

To satisfy the maximum feature size condition (Section 2.1) for the predicted operating wavelength of *λ *= 1030 nm, the size of a single rod in the core of the fabricated top‐hat fiber should have a diameter smaller than 500 nm. Taking into account technological limitations (the size of the furnace on the optical tower and the optimum diameter of the rods from which the fiber preform is stacked), it was decided to develop a fiber with a square core on a hexagonal lattice with the size of 85 rods on the diagonal (**Figure** **5**a). The total number of rods of such a structure is composed is 5419. In this case, 2^5 419^ possible structures can be arranged from two types of glasses, which precludes using deterministic methods to find their optimal arrangement. Therefore, to find a suboptimal solution, we use an in‐house developed numerical code based on probabilistic Monte Carlo algorithms to numerically optimize the distribution of the rods (Figure 5c). Monte Carlo algorithms rely on repeated random sampling to obtain optimized solutions, and they are commonly used to solve complex engineering problems with multiple degrees of freedom.^[^ ^36^ ^]^

Since the desired fundamental mode in the form of a square top hat shows two‐fold axial symmetry, optimizing the whole structure is unnecessary, but some areas can be multiplied, and calculation time is reduced. Therefore, we have determined in the calculated area two subareas indicated by different colors (blue and yellow), as shown in Figure 5a. The yellow area includes only the set of rods in the structure's upper‐right quarter. The internal structure of these rods can be calculated and replicated in each of the four quarters according to two‐fold symmetry. The blue area in the center has been separated as an area where symmetry rules do not affect rod distribution. According to our previous experience related to the design of nanostructured fiber, this approach gives better results.^[^ ^23^, ^25^, ^26^ ^]^ The distribution of nanorods in the core's central region significantly influences fiber performance and intensity distribution in the guided mode. Introducing symmetry rules would limit the flexibility of the design in this critical area. In total, the area to be optimized consists of 880 rods: 843 rods in the yellow region and 37 rods in the blue region.

As discussed in the introduction, the best structures previously theoretically considered for guiding a top‐hat fundamental mode are M‐type optical fibers. Therefore, the numerical optimization process in our algorithm starts from the structure shown in Figure 5b, which is a section of a square frame. In subsequent optimization steps, one rod is randomly selected from the blue or yellow area (Figure 5a) and changed to a rod made from the other type of glass, according to Figure 5c. Next, using the finite element method (FEM, COMSOL Multiphysics), the intensity distribution in the fundamental mode is calculated for a fiber with the spatial structure of the core. The calculated modes have discrete eigenvalues and their intensity distribution corresponds to eigenvectors calculated as direct numerical solutions of the wave equation for eigenmodes with specific border conditions determined by a nanostructure of the fibers. The obtained intensity distribution is compared with the ideal top‐hat distribution following the formula, which represents a cost function in our algorithm:

(2) $$E=\sum_{x,y}{\left(TH\left(x,y\right)-FM\left(x,y\right)\right)}^{2}$$

where *TH_x,y_* is the local intensity in ideal top‐hat mode and *FM_x,y_* is the local intensity in the fundamental mode calculated for the considered structure. If the change of the rod material decreases the cost function (*E*), the modified structure is accepted, and the algorithm starts a new sequence. In the opposite case, a new sequence without changes in the structure is needed. The numerical optimization is carried out until the change of the rod material does not reduce the cost function in the successive number of steps that corresponds to half of the total number of rods in the structure.

The optimization procedure for positioning 880 rods required ≈20 h of calculation using a dedicated server (4‐core, i7‐7700 CPU). Most of the computing time is consumed for calculating intensity distribution in the guided mode performed in the linked COMSOL Wave Optics module, where the calculation of effective modes for input fibers with the nanostructured core is implemented.

The effective refractive index distribution in the optimal structure (Figure 5d) has some clear similarities to M‐type fibers, but they are not the same. The frame‐like structure is formed by high refractive index nanorods grouped at the borders of the core. The main difference is the accumulation of a large number of high refractive index rods in the corners of the structure. They are responsible for the local increase of the effective refractive index and, thus for the increase of light intensity and maintenance of sharp corners in the intensity pattern of the fundamental mode. The central part of the core is filled with distributed high refractive index rods, which introduce increased effective refractive index concerning the cladding with small local gradients. We note that the central region of the optimal structure (the blue area in Figure 5a) exhibits two‐fold symmetry, but this is a result of an optimization procedure without symmetry constraints, and in general, this is not always the case.

**Figure 6:**
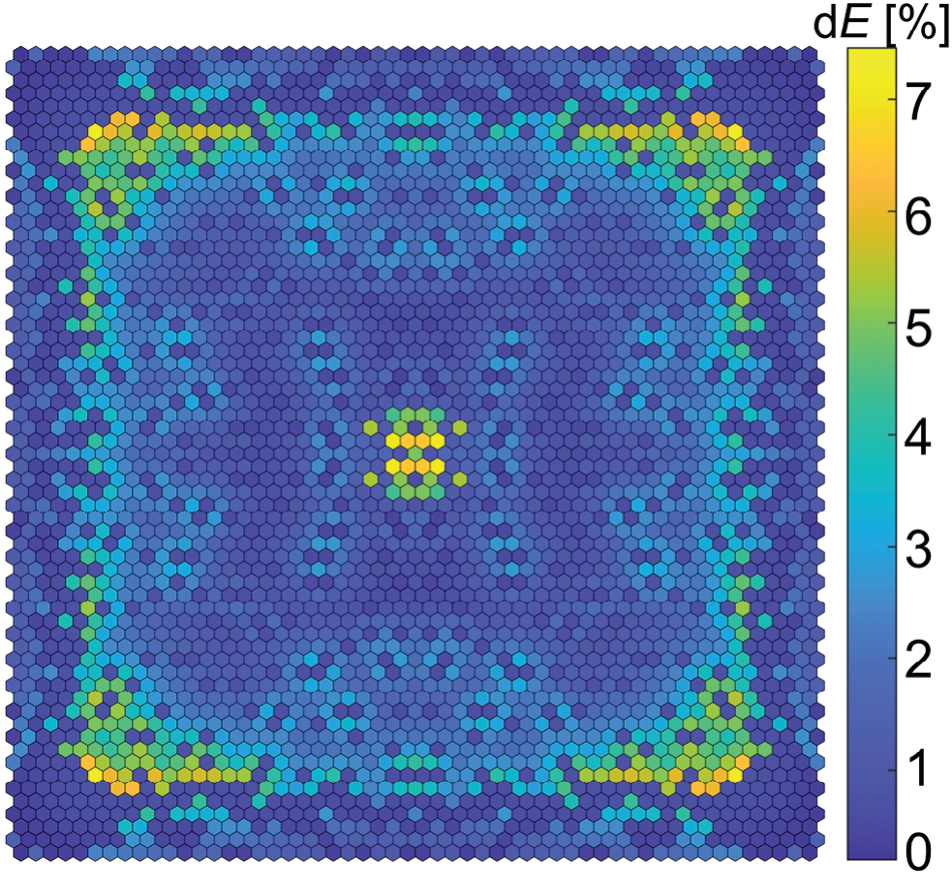
The cost function error map. An increase in the cost function is caused by using the wrong type of individual nanorod (high refractive index nanorod instead of low refractive index one, or opposite) in the fiber with optimized core nanostructure. An impact of error depends on the position of the incorrect nanorod in the fiber core structure.

The correct distribution of nanorods in a free‐form fiber is crucial to obtaining calculated optical properties. The calculated structure is sensitive to errors in nanorod distribution, but not all nanorods are equally important. We have calculated the influence of using the wrong individual nanorod (high refractive index nanorod instead of low refractive index one, or the opposite) on the cost function (**Figure** **6**). Most individual errors in using the correct rod type affect the cost function (Figure 2) by less than 4%. Rods located near the corners have a higher impact on the cost function of 6%. The most significant errors, up to 7%, occur when the wrong rod is located close to the center area.

**Figure 7:**
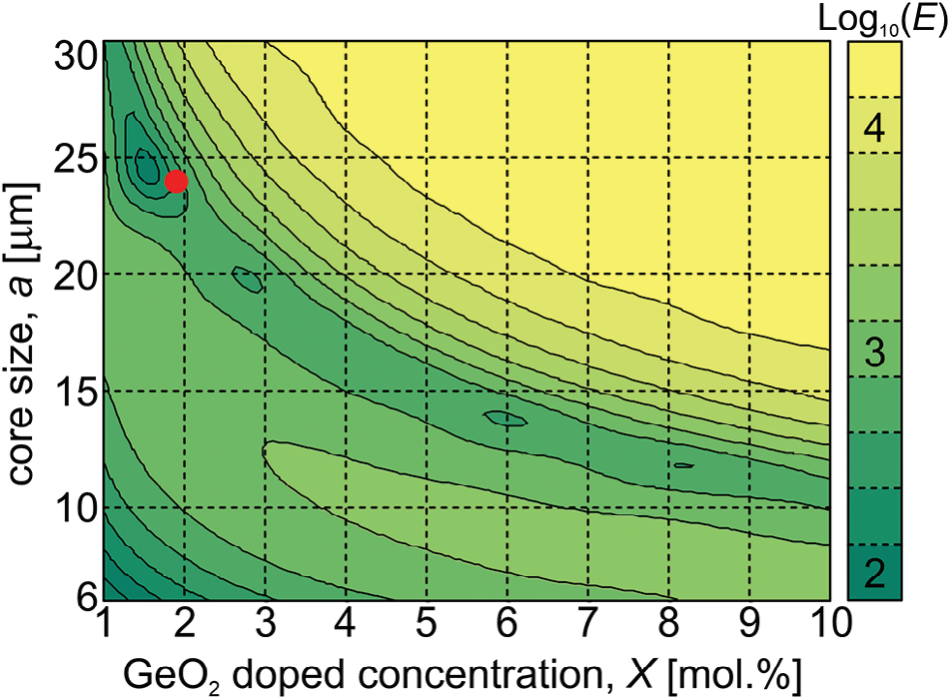
A quality reproduction of ideal square top‐hat intensity distribution in the fundamental mode in nanostructured free‐form fibers composed of pure silica and GeO_2_ doped silica rods for different core sizes a and the dopant concentration *X* in rods. The red point indicates the parameters of the structure that are evaluated experimentally (*X *= 1.9 mol.%, *a *= 24 µm).

**Figure 8:**
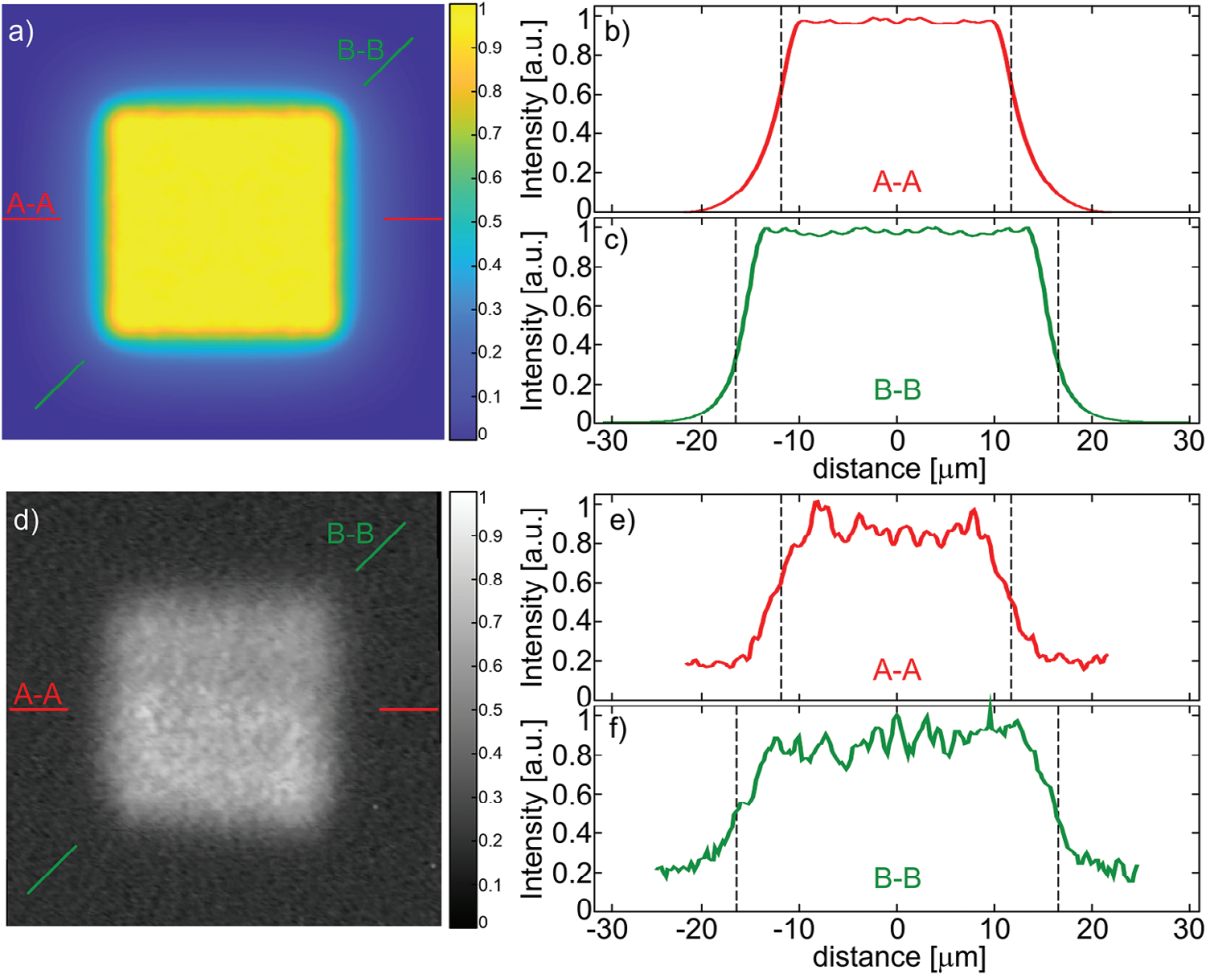
The intensity distribution of fundamental mode formed by optimized nanostructured free‐form fiber with a core of 24 µm and GeO_2_ doping of 1.9 mol.%. Numerical results: a) intensity distribution, b) cross section in A‐A direction, c) cross section in B‐B direction (diagonal). Experimental results: d) intensity distribution in the fiber measured as a near‐field intensity distribution at the face of the fiber, e) cross section in A‐A direction, f) cross section in B‐B direction (diagonal). The black dashed vertical lines indicate the boundary of the nanostructured core.

The optimization was carried out for a particular core structure with fixed core size a and the GeO_2_ doping level *X* of the rods. Any change in the above parameters influence significantly the calculated final nanostructure of the core and its cost function *E*. Optimization procedure was repeated for various parameters of doping level of GeO_2_ doped silica rods in the range 1–10 mol.% and size of a core in the range 6–30 µm. We have obtained different rod distributions in core structure and cost functions *E* for various sets of input parameters. The results presented in **Figure** **7** show that the most top‐hat‐like intensity distribution in the fundamental mode can be obtained for a structure with *a *= 25 µm and doping *X *= 1.5 mol.%. Due to the available preform doped with GeO_2_ at 1.9 mol.%, a fiber with a core with a side length of 24 µm was fabricated. This selection of parameters and the associated refractive index distribution provides a uniform light intensity distribution with a standard deviation of *σ *= 0.0096 (**Figure** **8**). The edges of the flat distribution region are not distorted, and the corners are only slightly rounded, but no intensity degradation is observed.

The final optimized structure consists of 5419 rods ordered into the hexagonal lattice to form a square core with 55 × 55 elements on their sides (Figure 5d). The core size is 24 × 24 µm and corresponds to an effective mode area of 360 µm^2^. The structure contains 1036 rods of *X *= 1.9 mol.%, GeO_2_ doped silica glass. The remaining rods are of pure silica glass.

Notably, the cost function (Equation (2)) used in optimizing the fiber structure can be modified for other, often opposing, requirements. A correct cost function can be defined as a weighted sum of several components of the cost function directly related to selected optimized parameters of the fiber. In the case of the fibers dedicated to guiding a mode with top‐hat intensity distribution, the cost function may include the components responsible for reducing the standard deviation of the intensity distribution, bending losses, confinement losses, or the presence of higher‐order modes.

## 3 Fabrication

**Figure 9:**
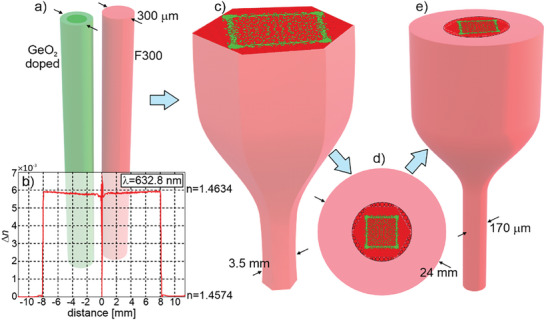
Scheme of the fabrication process of a nanostructured free‐form fiber: a) rods made from two types of thermally matched low and high refractive index glasses, b) cross section of refraction index distribution in the GeO_2_ doped silica rod used as high refractive index rods, c) stacking of the rods to form a core with the pattern according to the designed one and drawing a core sub‐preform, d) assembly of the final fiber preform, e) drawing of the final free‐form nanostructured fiber.

Considering the simulation results from Section 2 and the available materials, a few‐mode fiber with a square core of 24 µm side was selected for fabrication. A modified stack‐and‐draw method was used to fabricate the nanostructured fiber.^[^ ^24^ ^]^ Two thermally matched glasses were used for the optimized structure. Cladding and undoped rods (**Figure** **9**a) were fabricated from silica glass F300 (Heraeus). As high refractive index rods, we used an MCVD‐manufactured GeO_2_ doped silica preform rods (YOFC, China) with a flat refractive index distribution and central dip, as shown in Figure 9b. The effective doping of the rods is 1.9 mol.%, considering that the diameter of the whole preform is 22.8 mm and the diameter of the doped region is 16 mm. Two types of rods (Figure 9a) with diameters of 300 µm were drawn at the fiber drawing tower, cut to a length of ≈125 mm, and further assembled manually in the nanostructured preform according to the calculated pattern (Figure 5d). The fiber preforms are assembled manually, where individual rods are positioned layer by layer. This approach is sufficient for producing prototype fibers but not mass production. For practical applications, it would be necessary to use robotic assembly or direct 3D printing of glass preforms, as recently reported by a few groups.^[^ ^37^, ^38^, ^39^, ^40^ ^]^

**Figure 10:**
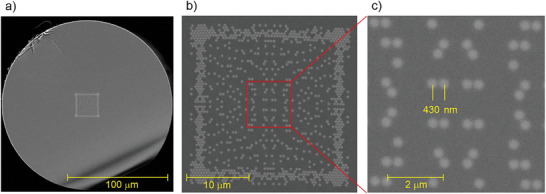
SEM images of the fabricated free‐form fiber with 24 µm nanostructured core: a) whole fiber, b) enlarged core region, c) central core area. The brighter areas correspond to the presence of germanium‐doped silica glass nanorods.

The preforms are assembled in dedicated chambers with laminar air flow equipped with ionized air suppliers. Next, the preform was integrated and scaled down to form a sub‐preform with a diameter of 3.5 mm in the subsequent fiber drawing process (Figure 9c). In the next step, the subpreform was placed into an F300 glass capillary with an outer diameter of 24 mm (Figure 9d). The spaces between the capillary and the core subpreform were filled with undoped glass rods. In the final step, the whole structure was drawn on an optical tower (Figure 9e). As a result, ≈1000 meters of a 170 µm diameter fiber with a 24 µm square core was obtained, which corresponds to a yield of 25%. SEM images of the core area confirm the correct distribution of nanorods concerning the design pattern and lack of significant diffusion (**Figure** **10**). The size of individual nanorods in the core is ≈430 nm, which meets the maximum feature size condition of the effective medium. The diameter of the GeO_2_ doped area is 340 nm. It is larger than the directly scaled value (305 nm) from the preform parameters due to the germanium diffusion process that occurs during fiber drawing. The diffusion process is similar for all nanorods in the fiber core (Figure 10b), and an additional 17 nm of ring thickness corresponds to the germanium diffused area.

We note that the fabricated proof‐of‐concept fiber has parameters close to the optimum one, as shown in Figure 7 (the core side length of 25 µm and effective nanorods doping of 1.5 mol.%) but not the same because we have used preform rods with the effective doping level of 1.9 mol.%, which is higher than optimum one.

## 4 Experimental Verification of the Optical Properties

The optical properties of the fabricated fiber were verified experimentally. A 1030 nm femtosecond laser with beam quality M2 = 1.20 (Menlo Systems, Blue Cut) was used as a source. A ×20 microscope objective (NA = 0.4) was used for laser beam coupling. The near field at fiber output was registered on a CMOS camera detector (Sony IMX179).

**Figure 11:**
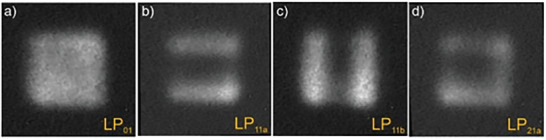
Experimentally observed in near‐field guided modes in a nanostructured free‐form fiber with a 24 µm core for the wavelength of *λ *= 1030 nm.

According to simulations, we predicted that the fabricated fiber guides 6 modes of 4 mode types (LP_01_. LP_11_, LP_21_, and LP_02_)when excited with 1.03 µm. Modes LP_01_. LP_11_ and LP_21_ were observed when they were selectively excited, as shown in **Figure** **11**. The high‐order LP_21b_ mode could not be excited in pure form.

An analysis of the contribution of eigenmodes to the formation of the output near‐field distribution measured experimentally was based on the mode decomposition method as previously proposed^[^ ^41^ ^]^

(3) $$P_{NF}\left(x,y\right)={\left|\sum_{l,m}C_{l,m}\Psi_{l.m}\left(x,y\right)\right|}^{2}$$

where *P_NF_* is the experimentally measured intensity distribution in the near field, Ψ*_l,m_* is the computer‐generated distribution of LP_l.m_ modes of the top‐hat fiber and *C_l,m_* is the weighting factors coefficient.

**Figure 12:**
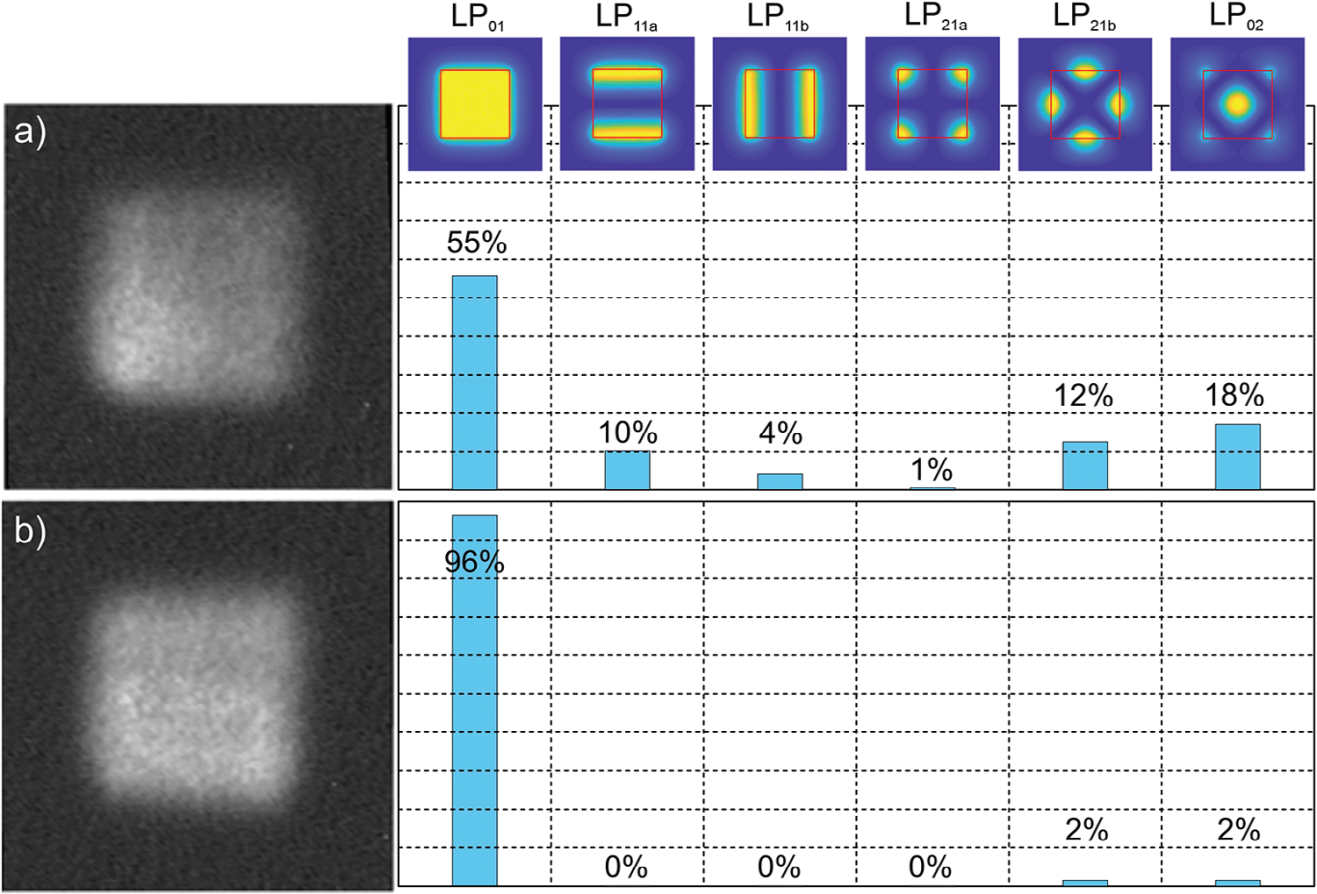
Measurements of mode contribution (in %) involved in forming the final near‐field light intensity distribution at the nanostructured fiber output: a) measured for a straight fiber, b) measured for a bent fiber (90° with a bending radius of 20 cm).

The mode decomposition analysis confirmed a guiding of 6 modes in the analyzed nanostructured fiber (**Figure** **12**), but the contribution of HOMs LP_11b_ and LP_21a_ in power balance is negligible. The measured purity of the fundamental mode is 55% in this case due to the high contribution of LP_11_, LP_21_, and LP_02_ modes (Figure 12a). A contribution of HOMs can be eliminated using the standard approach of fiber bending. We have shown that it is sufficient to bend the fiber by 90° with a bending radius of 20 cm to obtain effectively a single‐mode performance (Figures 8d and 12b). The measured purity of the fundamental mode increased to 96% with a small identified contribution of LP_02_ modes. We note that excitation of the fundamental mode is stable in this case, and changing free‐space laser coupling conditions by moving the fiber face concerning the collimating lens up to +/‐5 µm from the central position does not introduce any significant change in the observed mode purity. Also, further reduction of the bending radius of the fiber down to 10 cm does not cause substantial changes in the intensity distribution in the mode or mode purity. Further bending was impossible to verify experimentally due to the break of fiber, which was not protected by a polymer coating. We note that free‐form fibers can be polymer coated with standard single or multiple polymer layers when drawing on the optical tower, as with other silica fibers.^[^ ^24^, ^27^ ^]^ The obtained intensity distribution at the fiber output is characterized by a highly uniform light intensity distribution with a standard deviation of *σ *= 0.064 (Figure 8d–f).

The attenuation of the fundamental mode has been measured using the standard cut‐back method for a wavelength of *λ *= 1.03 µm for the samples of 2 m long. The total attenuation losses of 0.07 dB m^−1^ are measured. The measured value is relatively high but fully acceptable for laser machining systems where only a short section of fiber of a few meters is used. Losses are related to low numerical aperture NA = 0.03 of the fiber but mainly to material and fabrication conditions used during fiber manufacture. The quality of the germanium‐doped preform was limited. Also, the preform assembly and fiber drawing process was carried out in a standard research‐grade laboratory without high‐level cleanroom conditions. The fiber losses can be further reduced significantly if high‐quality optical rods are used and an industry‐standard clean room for preform assembly and fiber drawing is maintained.

**Figure 13:**
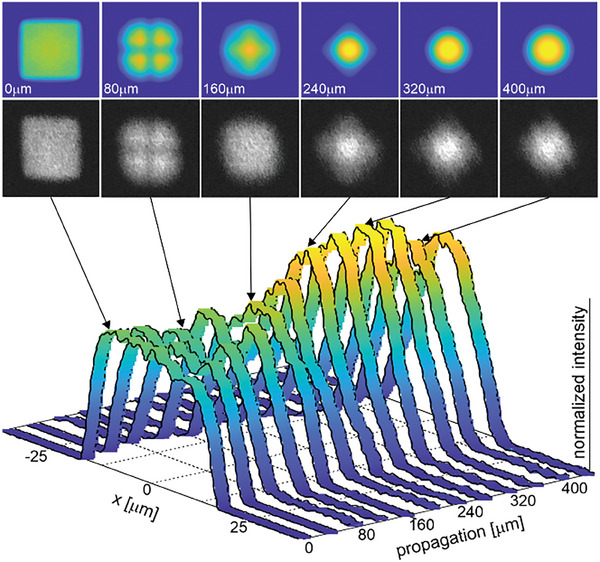
Evolution of light intensity distribution of square top‐hat mode during beam propagating in free‐space behind end face of the fiber in the function of the distance. The first row presents the results of numerical simulations calculated with the beam propagation method, and the second row and ribbon graph present matching experimental results.

We have also experimentally analyzed the evolution of beam properties during its propagation in free space behind the fiber (**Figure** **13**). The top‐hat distribution of the fundamental mode is observed on the face of the optical fiber (near‐field). The further evolution of the intensity distribution in the beam with increasing distance can be considered as Fresnel diffraction on a square aperture.^[^ ^42^ ^]^ Therefore, after ≈80 µm a darker cross can be seen in the image. Further propagation increasingly makes the intensity distribution similar to a Gaussian beam profile. The intensity distribution profile remains square even at 240 µm from the fiber end but rotated by 90°. The measurements agree with the numerical results using the beam propagation method (Figure 13) and beam propagation in the free space described by wave optics. A square mode with top‐hat intensity distribution can be maintained only in a guided medium as a waveguide with specific parameters. During free space propagation, the beam gradually transforms into a Gaussian beam at a distance of a fraction of a millimeter. We note that the intensity distribution profile presented in Figure 8d is different from the one presented in Figure 13. They come from different raw data since they were collected with two different imaging setups with different magnification and illumination conditions.

## 5 Conclusion

We have shown a new approach to effectively developing a large mode area, using single‐mode all‐solid fibers with flat top‐hat intensity distribution over the entire mode area and square cross‐section. For this purpose, we used a new approach to shape refractive index distribution in the core area using nanotechnology. The core area comprises a few thousand nanorods, either pure silica or germanium‐doped silica. Proper distribution of nanorods allows the shaping of a spatial mode profile and intensity distribution that is impossible with any other methods commonly used for optical fiber design.

We have shown that the design algorithm based on the Monte Carlo method leads to optimized nanorods free‐form distribution in the core that fulfills the requirements for the optical properties of the fiber. Based on the generated design, we fabricated all‐glass fiber with a square‐like core of 24 µm side, composed of 4387 silica glass rods and 1036 silica glass rods doped with germanium at a concentration of 1.9 mol.% using a modified stack‐and‐draw technique. The fabricated fiber is optimized for work at 1030 nm wavelength and effectively guides 4 modes with a dominant role of fundamental mode with a large mode area of 360 µm^2^, a square cross‐section, and top hat intensity distribution, as measured.

Experimentally, we verified a bend‐induced single‐mode performance of the fiber with a fundamental mode purity of 96%. This mode has a good homogeneity of light intensity over its entire square area with a standard deviation as low as *σ* = 0.064 without any significant changes in light intensity in the core corners, which was a drawback of previously considered M‐type fibers.^[^ ^7^, ^22^ ^]^

The free‐space excitation of the fundamental mode is stable, and no significant changes in the intensity distribution are observed for a +/‐5 µm misalignment of the collimation setup. The fiber has losses of 0.07 dB/m, which are entirely acceptable for laser machining systems, where only a short section of fiber of a few meters is used. Further reduction of fiber attenuation is feasible if high‐quality doped silica glass rods are used and clean‐room conditions for preform assembly are applied. The fiber parameters are optimized for 1030 nm femtosecond laser and their application for surface processing and waveguide writing, where uniform energy distribution and sharp edges are required.

Moreover, the high agreement between experimental results and numerical calculations indicates good control of the stacking and drawing process of the nanostructured fiber. The presented results also show the advantage of a new class of all‐solid fibers with nanostructured cores. Namely, free shaping of the effective refractive index distribution in the core is possible without rotational symmetry requirements. As a result, arbitrary profiles of modes and spatial intensity distribution can be obtained. A properly defined cost function can allow optimization of core nanostructure against contradicting fiber parameters, such as effective mode area, bending losses, and mitigation of higher‐order modes. We note that nanostructured fibers also have hard limits due to the material properties of glass nanorods, and in some cases, air‐glass fibers will offer better solutions for particular applications, such as multimode large‐mode‐area fibers.^[^ ^7^ ^]^

We note that further progress in technology and design methods is needed to take full advantage of free‐form fibers and their flexibility in shaping their optical properties. The manual assembly of fiber preform (assembly of a few thousand rods to form a preform) is time‐consuming and sensitive to human errors. It can be replaced with 3D printing of fiber preforms. This technology has been explored intensively recently.^[^ ^37^, ^38^, ^39^, ^40^ ^]^ Design of free‐form nanostructured fibers with deterministic algorithms is time‐costly, and only a limited number of parameters can be taken into account during optimization because the number of permutations in the considered core structures is 2^X^, where X denotes the number of nanorods in the fiber core (5419 rods in the case of the top hat fiber). Therefore, deep learning algorithms can be applied in the future for this purpose to optimize all critical fiber parameters (modal properties, intensity distribution in the guided modes, effective mode area, confinement, and bending losses), as we have recently presented preliminary results.^[^ ^43^ ^]^

## Conflict of Interest

The authors declare no conflict of interest.

## Data Availability

The data that support the findings of this study are available from the corresponding author upon reasonable request.
